# Development of pH-Responsive Biopolymeric Nanocapsule for Antibacterial Essential Oils

**DOI:** 10.3390/ijms21051799

**Published:** 2020-03-05

**Authors:** Sylvie Skalickova, Tereza Aulichova, Eva Venusova, Jiri Skladanka, Pavel Horky

**Affiliations:** Department of Animal Nutrition and Forage Production, Faculty of AgriSciences, Mendel University in Brno, Zemedelska 1, 613 00 Brno, Czech Republic; tereza.aulichova@mendelu.cz (T.A.); eva.venusova@mendelu.cz (E.V.); jiri.skladanka@mendelu.cz (J.S.); pavel.horky@mendelu.cz (P.H.)

**Keywords:** alginate, chitosan, *E. coli*, guar gum, gastro-intestinal tract, pectin, *Rosmarinus officinalis*, *S. aureus*, *S. cerevisiae*, *Syzygium aromaticum*, *Thymus vulgaris*, xanthan gum

## Abstract

It is generally believed that antibacterial essential oils have the potential to become one of the alternatives in preventing diarrheal diseases of monogastric animals. The disadvantage is their low efficiency per oral due to easy degradation during digestion in the stomach. This study compares the efficacy of chitosan, alginate-chitosan, guar gum-chitosan, xanthan gum-chitosan and pectin-chitosan nanocapsules to the synthesis of pH-responsive biopolymeric nanocapsule for *Thymus vulgaris*, *Rosmarinus officinalis* and *Syzygium aromaticum* essential oils. Using spectrophotometric approach and gas chromatography, release kinetics were determined in pH 3, 5.6 and 7.4. The growth rates of *S. aureus* and *E. coli*, as well as minimal inhibition concentration of essential oils were studied. The average encapsulation efficiency was 60%, and the loading efficiency was 70%. The size of the nanocapsules ranged from 100 nm to 500 nm. Results showed that chitosan-guar gum and chitosan-pectin nanocapsules released 30% of essential oils (EOs) at pH 3 and 80% at pH 7.4 during 3 h. Similar release kinetics were confirmed for thymol, eugenol and α-pinene. Minimal inhibition concentrations of *Thymus vulgaris* and *Syzygium aromaticum* essential oils ranged from 0.025 to 0.5%. Findings of this study suggest that the suitable pH-responsive nanocapsule for release, low toxicity and antibacterial activity is based on chitosan-guar gum structure.

## 1. Introduction

Antibiotics have been used as effective drugs for several decades. Livestock farming is one of the sectors where antibiotics had been overused. Since 2006, the European Union has prohibited the use of antibiotics as growth promoters, and new alternatives are sought to maintain high livestock production [1]. Essential oils are often discussed as a suitable alternative. Thyme, oregano, clove, cinnamon and tea tree are considered the most effective essential oils (EOs) in terms of antimicrobial activity in vitro and in vivo. Their mechanism of action is based on the damage or increase of permeability of bacterial cell wall, disruption of the bacterial film, interruption of the ATP production pathway and inhibition of proteosynthesis [2]. The antioxidant activity of some oils and the proven genoprotective effects are also significant. On the other hand, some EOs has been considered as toxic or genotoxic on the eucaryotic organism. Therefore, their use should undergo a toxicological evaluation [3]. Their effects depend most on their chemical composition. Generally, EOs consist of aromatic and aliphatic compounds, hydrocarbon terpenes (isoprenes) and terpenoids (isoprenoids). The proportion of individual substances varies due to the extraction method and storage conditions [4]. A number of studies are in good agreement that the effects of EOs are influenced by their degradation rate and bioavailability in the gastrointestinal tract of animals. The digestive processes respectively reduce their effect and increase the doses [5].

In order to protect EOs from degradation, one of the suitable alternatives is encapsulation into biopolymeric microparticles or nanoparticles [6]. Generally, biopolymeric (nano)particles could be based on proteins, lipids or polysaccharides. The polysaccharide chitosan (C) has been dominant in this area for several years. C is prepared from chitin shells of crustaceans, which are actually the waste. The absolute advantages are the ease of (nano)material preparation, low cost and possibility of large-scale production. Due to popularity, C is well examined both in material science and applications in pharmaceutical and molecular studies. Chitosan has bioadhesive, biocompatible properties, stimulates the immune system, and is biodegradable and low in toxicity. Moreover, chitosan shows antifungal and antimicrobial effects against both G+ and G− bacteria [7]. The main properties for some polysaccharide-based biopolymers being sought out is the resistance against enzymatic digestion in the oral cavity and low pH in the stomach, but they are easily degradable in higher pH or by the intestinal microflora. This option makes them suitable for EO delivery to the intestines and colon. Together with some other polysaccharides such as pectin, alginate, guar gum, xanthan gum and dextran, they very readily form nanogels, nanoemulsions, nanoparticles or nanocapsules [8]. Several methods have been described for (nano)particle, (nano)gel and (nano)emulsion synthesis. Generally, emulsification, coacervation, polymerization and crosslinking are based on good homogenization and the addition of various excipients. In contrast, physical procedures such as electrospray or electrospinning require special equipment and devices [9,10].

The aim of this research is to encapsulate EOs into chitosan-polysaccharide nanocapsules (C-NCaps). Our study is focused on the evaluation of various NCaps composites for pH-responsive release of EOs. From our best knowledge, a comprehensive comparison of the impact of chitosan, alginate, xanthan gum, guar gum and pectin in this field of research is still missing.

## 2. Results

### 2.1. Effect of TPP and Tween 80 on Nanocapsules EE and LE

The procedure of C-NCap formation was adapted to the conditions of large-scale production and to produce easy and cost-effective technology. The procedure consists of two steps: 1. EOs emulsification in the polysaccharide aqueous phase (C-C, C-A, C-G, C-X and C-P) and 2. crosslinking the structure via tripolyphosphate (TPP). Optimization steps include monitoring the influence of Tween 80 and TPP concentration on the encapsulation efficiency (EE). *Thymus vulgaris* (T-EOs), *Syzygium aromaticum* (S-EOs) and *Rosmarinus officinalis* (R-EOs) of various concentrations (0.01–1.5%) were encapsulated, and the loading efficiency (LE) was calculated. Evaluation parameter also included accessibility of the chitosan amino groups, which were determined by the ninhydrin reaction. From our previous results, the higher signal indicates a looser crosslinking of nanostructures [11].

**Figure 1:**
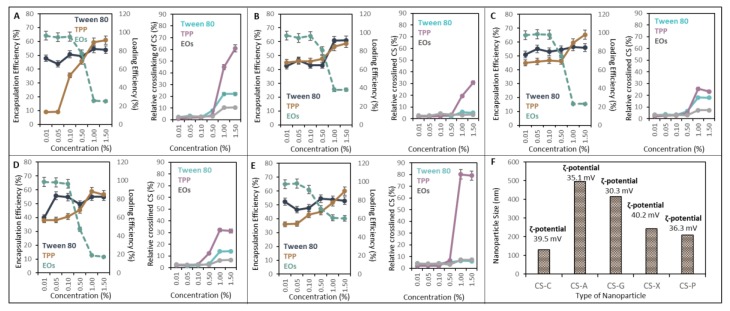
The optimization of NCap preparation: (**A**) C-C, (**B**) C-A, (**C**) C-G, (**D**) C-X and (**E**) C-P NCaps. The tested concentrations of Tween 80, tripolyphosphate (TPP) and essential oils (EOs) were in the range from 0.01 to 1.50% of the total volume of the reaction mixture. Figures on the left: encapsulation efficiency is calculated as the total concentration of EOs related to encapsulated concentration EOs in NCaps. LE (secondary axe, dashed line) is calculated as a total concentration of EOs related to NCap weight. On the right: relative crosslinking of C under various TPP, Tween 80 and EOs concentrations. (**F**) Size and ζ-potential of the prepared Ncaps: Results are expressed as an average of three measurements *n* = 3.

The optimization of C-NCaps formation was performed with S-EOs, which gives the most sensitive signal response. The strongest influence of TPP on EE showed C-NCaps. From Figure 1A, the strong EE increase depending on the increase of TPP concentration is obvious. For other variants of NCaps, the EE varies from 40 to 60% depending on the TPP concentration, whereas high EE was achieved from the 1% TPP concentration (Figure 1B,C).

Tween 80, as an emulsifier, showed a moderate influence on EE in the cases of C, C-G, C-X and C-P NCaps (Figure 1A,C–E). The concentration of 0.5% Tween 80 increased the EE of C-A Ncaps by 30% (Figure 1B). LE (dashed) assessment proved that 0.1% of EOs are encapsulated from 65% in all variants (Figure 1A–E). Higher applied concentrations of EOs (1–1.5%) showed LE around 20% except for C-P NCaps, where even at higher concentrations, LE was observed at 40% (Figure 1E). When assessing the degree of crosslinking of the capsule structure, it is evident that the addition of TPP has the greatest effect over Tween 80 (Figure 1A–E). The most pronounced effects were observed in C and P NCaps. EOs did not affect the crosslinking of the structure.

Dynamic light scattering was used to measure the size of the NCaps. C-NCaps diameter was 100 nm. It can be seen that, in combination with other polysaccharides, the capsule size increases. The most pronounced effect was observed in C-A NCaps, where the particle size reaches more than 500 nm. Similarly, C-G NCaps showed a size of around 400 nm. The C-X and C-P NCaps had dimensions of 300 nm. The measured ζ-potential varies from +30 to +40 mV (Figure 1F). The particles have a cationic character and are stable in the colloidal system.

### 2.2. EOs Release from NCaps under Physiologic Conditions

**Figure 2:**
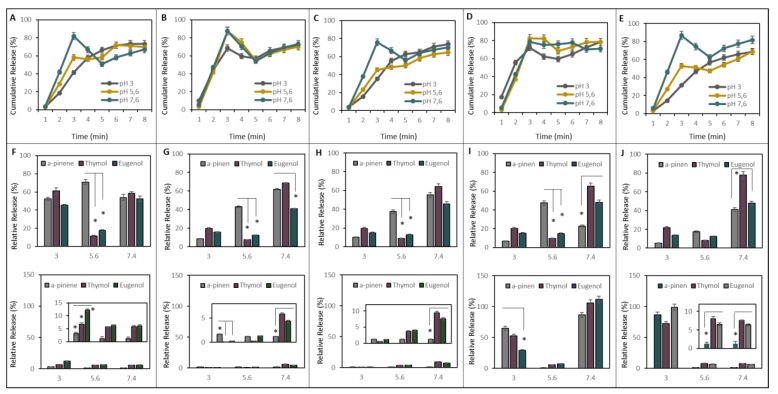
The release study: All types of NCaps and (0.1%) EOs were tested in pH 3, 5.6 and 7.6 during 8-h incubation under 37 °C. The cumulative release of (**A**) C, (**B**) C-A, (**C**) C-G, (**D**) C-X and (**E**) C-P NCaps were examined. The relative release of active substances (thymol, eugenol and α–pinene) from (**F**) C, (**G**) C-A, (**H**) C-G, (**I**) C-X and (**J**) C-P NCaps after 1 h is shown on the top and after 8 h is shown on the bottom. The asterisks indicate a difference in the significance level *p* < 0.05. Results are expressed as an average of three measurements *n* = 3. The asterisks indicate a difference in the significance level *p* < 0.05.

The cumulative release of EOs was investigated using S-EOs, which gave the most sensitive signal response with Folin-Ciocalteau (FC) reagent. Unbound EOs were washed away from the NCaps using a three-kDa cutoff filter. Its pores enable free EOs to pass through the membrane, but NCaps were trapped. The graphs in Figure 2 show that there is rapid release in the first 3 h for all variants. However, EOs release is significantly higher at pH 7.4 than at pH 3 and 5.6 in C, C-G and C-P NCaps (Figure 2A,C,E).

We monitored the release of the active substances of EOs: thymol (T-EOs), eugenol (S-EOs), and α-pinene (R-EOs) by gas chromatography with flame ionization detection (GC-FID). The results are expressed as the amount (%) of the active substances released at pH 3, 5.6 and 7.4 during the first hour and eight hours of incubation. From the graphs (Figure 2F–J top), it is apparent that thymol and eugenol were significantly less released at pH 5.6 compared to pH 3 and 7.4 in all NCap variants except C-P NCaps. Eugenol was slightly released at pH 7.4 from C-A NCaps (Figure 2G top) compared to thymol and α-pinene. α-pinene was significantly less released at pH 7.4 of C-X NCaps (Figure 2I top). Thymol was rapidly released at pH 7.4 from C-P NCaps (Figure 2J top). Comparing the individual variants of NCaps, it could be assumed that C-NCaps release its cargo readily at pH 3 and 7.4 (Figure 2F top). For the other variants, the higher release at pH 7.4 is predominant, at an average of about 70%, which is 3.5 times more than at pH 3. The release results of the active substances obtained after 8-h (Figure 2F–J bottom) incubation show significantly lower relative release instead of C-X and C-P NCaps. In these cases, the percentage of released active substances is more than 20% higher compared to the first hour at pH 3 and 7.4 (Figure 2D,E). Compared to the release of the active ingredients, thymol, then eugenol and, most poorly, α-pinene were released.

### 2.3. Inhibition Activity on Eucaryotic Model and Blood Compatibility Assessment of NCaps

**Figure 3:**
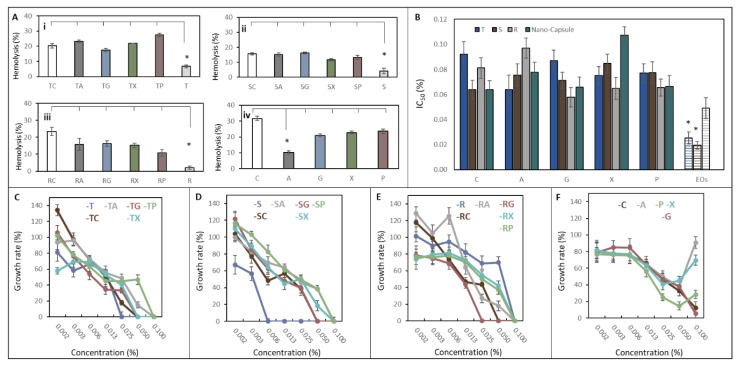
Inhibition activity on eucaryotic model and blood compatibility assessment of Ncaps: (**A**) Hemolytic assay of C-NCaps (concentration of EOs was 0.1% *v/v*): (**i**) *Thymus vulgaris*, (**ii**) *Syzygium aromaticum*, (**iii**) *Rosmarinus officinalis* and (**iv**) empty NCaps. As a positive control, 30% H_2_O_2_ was used, and the negative control was phosphate buffer saline (PBS). (**B**) Comparison of IC_50_ of studied NCaps and EOs (*Thymus vulgaris* (T), *Syzygium aromaticum* (S), *Rosmarinus officinalis* (R) and empty Ncaps). The growth rate of *S. cerevisiae* in the presence of EOs *Thymus vulgaris* NCaps is shown for (**C**) *Thymus vulgaris*, (**D**) *Syzygium aromaticum,* (**E**) *Rosmarinus officinalis* and (**F**) empty NCaps. The growth rate was calculated as a percentage of growth rate from untreated *S. cerevisiae*. Results are expressed as an average of three measurements *n* = 3. The asterisks indicate a difference in the significance level *p* < 0.05.

In terms of blood compatibility assessment, the results indicate that EOs alone show a low percentage of haemolysis compared to loaded NCaps or empty NCaps (Figure 3A). T-loaded NCaps and NCaps without EOs show the highest hemolytic activity (Figure 3Ai). There were no significant differences between NCaps, except for the low hemolytic activity of empty C-A N-Caps.

IC_50_ of *S. cerevisiae* ranged from 0.06 to 0.09% for encapsulated variants. IC_50_ was significantly reduced in T-EOs and S-EOs to 0.025% and 0.019%, respectively (Figure 3B). Furthermore, the growth rate from growth curves was evaluated. There was no significant difference in *S. cerevisiae* growth rate for T-EOs and R-EOs and its encapsulated variants (Figure 3C–F). The significant difference was observed only in the sample of S-EOs. *S. cerevisiae* did not grow at concentrations higher than 0.0063%. Concentrations of NCaps alone significantly affect the growth rate of the *S. cerevisiae* (Figure 3F).

### 2.4. Inhibition Effect of NCaps on G+ and G− Pathogenic Bacteria

**Figure 4:**
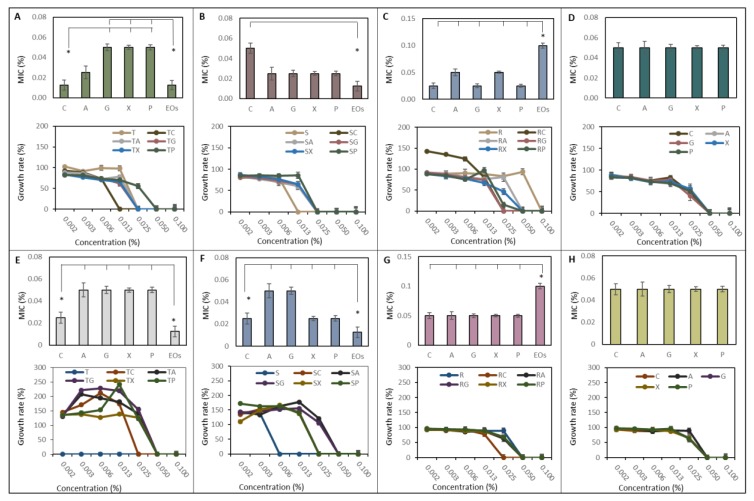
Inhibition activity of NCaps (C-C, A-C, G-C, X-C, P-CS and EOs (0.002–0.1% *v/v*)). The inhibition effect on *S. aureus*: (**A**) T-EOs, (**B**) S-EOs, (**C**) R-EOs and (**D**) NCaps alone. The inhibition effect on *E. coli*: (**E**) T-EOs, (**F**) S-EOs, (**G**) R-EOs and (**H**) NCaps alone. The minimal inhibition concentration (MIC) is shown on the top, and growth rate is shown at the bottom. Results are expressed as an average of three measurements *n* = 3. The asterisks indicate a difference in the significance level *p* < 0.05.

*S. aureus* and *E. coli* were chosen as representatives of pathogenic bacteria. The influence of T-EOs and its encapsulated variations on *S. aureus* is shown in Figure 4A. The minimal inhibition concentration (MIC) of T-EOs NCaps varies in the range of 0.01 to 0.05%, whereas C-NCaps and T-EOs MIC were significantly decreased compared to other variants. The applied concentration of the treatment influences the growth rate of *S. aureus* but, among NCaps variants, is consistent. S-EOs NCaps did not show a significant difference of MIC (0.05%) in all studied variants (Figure 4B). However, the MIC of S-EOs was significantly lower than the MIC of C-NCaps. Obtained growth rates did not show significant differences between S-EOs NCaps. R-EOs showed significantly higher MIC (0.1%) compared to its encapsulated form (0.05%) (Figure 4C). Growth rates of *S. aureus* treated by R-EOs C-NCaps showed the highest growth rate at low concentrations. The R-EOs has a constant influence on *S. aureus* growth rate up to a concentration of 0.05%, confirming the results of the MIC. Inhibition influence of empty NCaps is shown in Figure 4D. Both average MIC (0.05%) and growth rates indicate a similar effect among tested variants.

*E. coli* showed significantly increased sensitivity on T-EOs. The obtained MIC was in the range from 0.025 to 0.05% (Figure 4E). The MIC of T-EOs C-NCaps, C-X NCaps and C-P NCaps were 0.025%, which was significantly less compared to other variants of T-EOs NCaps. The bacterial growth rate varied between 150% and 250% for the tested variants, with maximum concentration values about 0.025%. Similarly, as T-EOs, the S-EOs showed a significantly increased antibacterial activity against *E. coli* with MIC 0.0125%. The results are also confirmed by the growth rate, which increases to 150% at a concentration of 0.003% (Figure 4F). R-EOs NCaps and R-EOs, as well as NCaps alone, did not show significant differences in MIC and growth rates (Figure 4G,H).

## 3. Discussion

C has a wide variety of modification possibilities, which are due to the presence of reactive amino groups in its molecule. Similar studies confirm the strong effect of the crosslinking agent on crosslinking rates in ionotropic gelation processes. Also, the current study found that a higher concentration of Tween 80 as an EO emulsifier showed a weak effectivity on EE and LE but could affect the level of crosslinking [12,13]. Moreover, the emulsification and the diameter of oil droplets has shown a direct effect on particle size and encapsulation efficiency [14].

The active substances are volatile, and it is, therefore, important to monitor their release. In the current study, comparing the release ratio among C-NCaps, G-NCaps and P-NCaps has shown the different cumulative releases of encapsulated EOs at acidic and basic pH. Contrary to expectations, this study did not find a significant difference between C-A and C-X NCaps on pH-dependent release. However, most studies have verified the stability of alginate and xanthan at high pH [15,16,17]. You et al. determined a 40% release of C-A particles after 4 h in pH 7.4 and a 20% release of haemoglobin in pH 4.4 [18]. Kulkarni et al. demonstrated a 40–50% release of glipizide from C-X gum beads [19]. In accordance with the presented results, previous studies have demonstrated the increased release of cargo from G and P (nano)particles. The level of active substance release obtained after 8 h of incubation showed significantly lower relative release except for C-X and C-P NCaps. In this case, the release of active substances is on average 20% higher compared to the first hour for pH 3 and 7.4 for C-X and pH 3 for C-P NCaps. A possible explanation for these results may be the moderate rate of swelling of xanthan gum and pectin [20,21,22,23]. Compared to the release of the active substances alone, thymol was the best, then eugenol and then α-pinene was the worst in terms of release.

Saccharomyces cerevisiae is a commonly used eukaryotic model microorganism with many advantages such as simple structure, fully interpreted genetic background and ease of manipulation. *S. cerevisiae* has been used as a model for detecting drug exposure-associated toxic effects to quickly provide functional clues and to pave the way for more complex studies in animals or humans [24]. In our study, we tested the viability and growth rate of *S. cerevisiae* in the presence of EOs and NCaps. Obtained IC_50_ was significantly reduced for the *Thymus vulgaris* and *Syzygium aromaticum* EOs but not for Rosmarinus officinalis. Although studied EOs are generally regarded as safe, some publications have confirmed our results. Kunicka-Styczyńska noted the minimal inhibition concentration for *S. cerevisiae* of thyme EO was 0.5 µg/mL [25]. Konuk et al. published an MIC of *Thymus vulgaris* EOs at 0.2–0.3 µL/mL, but no inhibition activity was estimated in the cases of *Syzygium aromaticum* and *Rosmarinus officinalis* oils [26]. Konuk assumed the toxicity mechanism is due to the intracellular ions leakage through the cell membrane. Furthermore, it is obvious that the NCaps themselves showed low inhibition activity and reduce the toxicity of the encapsulated oils. In this case, the effect of EOs may be associated with their release depending on the pH of the environment. These results are also confirmed by the growth curves and calculated growth rates. NCaps have an effect on the growth rate of yeast. It is estimated that the growth rate increases in correlation with the concentration of empty NCaps. Surprisingly, no similar effect was observed in other publications. Chitosan cannot be utilized by yeast and, in higher concentrations, causes cell membrane leakage at concentration 15 µg/mL and inhibits cell growth in the range from 0.1 to 2 mg/mL [27,28]. However, it has been shown that *S. cerevisiae* has two chitin deacetylase genes, CDA1 and CDA2, that are transcribed only during sporulation and are not necessary for viability [29]. The blood compatibility assessment showed increased hemolytic activity of NCaps. De Lima et al. observed that hemolytic activity depends on the pH of the environment, whereas a neutral environment was reducing hemolysis [30]. More than hemolytic, chitosan is agglutinating, supported by wound healing studies [31].

The results show that the non-encapsulated EOs are more effective or exhibit the same antimicrobial activity as encapsulated EOs. In our study, these findings have been expected and are consistent with other studies [32,33,34]. On the other hand, it must be mentioned that some researchers have achieved the opposite results [35]. In our case, we assume that the lower antibacterial effect of EOs-NCaps is due to the release kinetics, which supports the conclusions of other researchers. In contrast, *Rosmarinus officinalis* EO did not show increased antibacterial effects over NCaps alone. It is known that the inhibitory effect of *Rosmarinus officinalis* is the result of the action of the composition of active substances. They interact with the cell membrane, causing changes in genetic material and nutrients, altering the transport of electrons, leakage of cellular components and production in fatty acid [36]. Thus, the low inhibitory effect of *Rosmarinus officinalis* EOs may be due to the low concentration of the active ingredients. Comparing NCaps themselves, no significant differences are apparent in the inhibitory activity but the inhibition is not evident (0.05%). This phenomenon is probably due to the antibacterial activity of chitosan itself [37].

## 4. Materials and Methods

### 4.1. Chemicals and Reagents

Low molecular weight chitosan (C), acetic acid, Folin-Ciocalteau reagent, Na_2_HCO_3_, Sodium Tripolyphosphate penta basic (TPP), ninhydrin, hydrindatin, dimethyl sulfoxide (DMSO), Tween 80, Sodium Acetate, eugenol, a-pinene, thymol and other chemicals unless noted otherwise were purchased from (Sigma Aldrich St. Louis, USA). *Thymus vulgaris*, *Syzygium aromaticum* and *Rosmarinus officinalis* were purchased from (doTERRA, Pleasant Grove, USA). Alginate (A), Xanthan Gum (X), Guar Gum (G) and Pectin (P) were purchased from (Fichema, Brno, Czech Republic). *S. cerevisiae* (ATCC 9763), *S. aureus* (ATC 25923) and *E. coli* (ATCC 25922) were obtained from the Czech Collection of Microorganisms, Faculty of Science, Masaryk University, Brno, Czech Republic. The pH value was measured using inoLab Level 3 (Wissenschaftlich-Technische Werkstatten GmbH; Weilheim, Germany). Deionised water underwent demineralization by reverse osmosis using the instruments Aqua Osmotic 02 (Aqua Osmotic, Tisnov, Czech Republic).

### 4.2. Biopolymeric EOs-NCaps Preparation

EOs-NCaps were synthesized by the ionic gelation method via interaction with TPP according to the method described in the literature with slight modifications [38]. Briefly, the procedure includes dissolving 0.3 g C in 100 mL acetic acid (1%) and subsequent neutralization (pH 6.7) by 1 M NaOH. A, X, P and G stock solutions were prepared by dissolving 3 g of each polysaccharide in 100 mL dH_2_O. All prepared solutions were incubated at 37 °C until clear on Orbital incu shaker (Biosan, Riga, Latvia); 1% EOs (or 0.05%, 0.1%, 0.5% and 1.5%) were dissolved in 200 µL aqueous solution of Tween 80 (0.05%, 0.1%, 0.5% and 1.5%). The mixture was sonicated ten minutes in ultrasonic bath (180 W, 40 kHz DU-45, Argolab, Carpi, Italy). Then, 300 µL of C stock solution was added and sonicated for 30 min. Subsequently, 300 µL of polysaccharide stock solution (A, X, P and G) was added and the mixture was vigorously shaken and sonicated for 30 min. Then, 300 µL of TPP (1 g in 100 mL of water) was added, followed by vigorously shaking and incubation in an ultrasound bath for 30 min. The mixture was centrifugated at 16,000 g Eppendorf centrifuge 5425 (Eppendorf, Hamburg, Germany), and the pellet was purified from the unbounded EOs by repeated centrifugation. Pellet and supernatant were taken for further analysis.

### 4.3. Folin-Ciocalteau Reaction

EOs from obtained the pellet and supernatant were quantified by Folin-Ciocalteau reaction (FC). The principle is based on the reaction of polyphenols (and reducing substances) contained in EOs with FC reagent [39]. The assay was modified into a microplate template; 10 µL of the sample was pipetted to 80 µL FC reagent (10 times diluted by dH_2_O). After 5 min of incubation, 100 µL of (0.1 M) Na_2_HCO_3_ was added. The absorbance at 700 nm was read after 10 min of incubation (22 °C).

### 4.4. Ninyhdrin Assay for Chitosan Detection

The method has been adopted and modified according to Reference [40]. The ninhydrin reagent was freshly prepared on the day of the assay by adding 25 mL of 4 M acetate buffer (pH 5.2) to 2 g ninhydrin and 0.3 g hydrindantin in 75 mL DMSO. For the assay, 75 µL of reagent was added to 100 µL of the sample in the Eppendorf tube. The microtubes were immediately capped, briefly shaken by hand and heat thermoblocked (Biosan, Riga, Latvia) for 30 min to allow the reaction to proceed. After cooling, 15 mL of a 50:50 ethanol:water mixture was added to each sample. The color intensity of the complex was spectrophotometrically evaluated (λ_max_ = 570 nm) as a measure of depolymerized C activity. Accurately weighed depolymerized chitosans were dissolved in 1% *w/v* acetic acid. A blank solution was also prepared in an identical manner, wherein 1% *w/v* acetic acid was used instead of a C solution to prepare the reaction mixture. The effect of the solvent system was multiplied by calibrating the instrument to 100% transmittance of the blank solution. Relative absorbance for each C-NCaps solution was calculated as follows:

RA = A of chitosan solution/A of standard solution(1)

### 4.5. Absorbance Measurements

The samples for measurements were placed in 96-well microtitration plates (Nunc™ MicroWell™ 96-Well Microplates, Thermofisher Scientific, USA). All measurements were performed at 22 °C on the Synergy HTX microplate reader Synergy HTX (Biotech, Minneapolis, MN, USA).

### 4.6. Encapsulation Efficiency and Loading Efficiency Calculation

The encapsulation efficiency (EE) of the C-S NCaps was expressed as the actual EO loading of the NCaps referred to the initial amount of EOs used. The encapsulated EO concentration was calculated from the calibration curve. EE (%) was calculated using the following equation:

EE (%) = (amount of EOs in NCaps/amount of initial EOs used) × 100(2)

The loading efficiency (LE) indicating the percentage of the mass of the nanocapsule that is due to the encapsulated drug.

LE (%) = (concentration of entrapped EOs/total nanocapsule weight) × 100(3)

### 4.7. Nanocapsule Size and ζ-potential Measurement

The average particle size and size distribution were determined by quasielastic laser light scattering with a Malvern Zetasizer NANO-ZS (Malvern Instruments Ltd., Malvern, United Kingdom). Nanocapsule distilled water solution of 1.5 mL (1 mg/mL) was put into a polystyrene latex cell and measured at a detector angle of 173°, a wavelength of 633 nm, a refractive index of 0.30, a real refractive index of 1.59 and a temperature 25 °C.

### 4.8. The Cumulative Release of EOs

Five hundred0 µL of NCaps were added into 4 (1.5 mL) tubes (Eppendorf, Hamburg, Germany). Each tube was filled with 500 µL of 10 mM PBS at pH 3, 5.6 and 7.4. Samples were incubated on an incubator shaker (Biosan, Riga, Latvia,) for 30 min at 30 rpm at 37 °C. Subsequently, a 100-µL aliquot was sampled and refilled with an equal volume of fresh buffer correspondingly. The aliquot of the sample was purified via 3k centrifugal filter (Amicon, Merck, Darmstadt, Germany), 4000 g (Eppendorf 5425, Germany). Filtrate and retentate were analysed. The process was repeated every hour until the end time of the analysis (8 h). The cumulative relative release was calculated according to the subsequent equation:

Relative release (%) = (encapsulated EOs concentration/released EOs concentration) × 100(4)

Cumulative release (%) = (relative release at time t − 1) + relative release at time t(5)

### 4.9. Gas Chromatography with Flame Ionisation Detection

Prior to the GC-FID analysis, the samples were extracted to n-hexane; 100 µl of the sample was vigorously shaken with 500 µL of n-hexane and incubated at 300 rpm at 10 °C (TS-100, Biosan, Latvia). The EO determination was performed using a GC system (Agilent 6890, Santa Clara, CA, USA) equipped with an FID. Separation of compounds was conducted on a 30 m Zebron ZB-WAX capillary column of 0.25 mm i.d. and 0.25 µm film thickness (Phenomenex, Torrance, CA, USA), using nitrogen 5.0 as the carrier gas (Siad, Rajhrad, Czech republic). The injection volume was 1 µL, and the flow rate was set on 0.7 mL/min. The injector temperature was 250 °C with a split ratio of 50:1, and the FID temperature was 220 °C. The oven temperature was programmed as follows: the column was held initially at 70 °C for 0.5 min, then increased to 190 °C at 20 °C/min and held for 4 min. Chromatographic data were recorded and integrated using Clarity software (Data Apex, Prague, Czech republic).

### 4.10. Minimal Inhibition Concentration Determination

50 µL of 5% DMSO was pipetted to the sterile 96-well microtitration plate Nunc (Thermofisher Scientific, Waltham, MA, USA). First columns were filled with 50 µL of tested samples in triplicates. Samples were diluted by half-dilution with a multichannel pipette (Eppendorf, Hamburg, Germany). The last column was left as a negative control. As a positive control, gentamycin (300 µg/mL) was used. The bacterial culture was diluted to the fresh sterile Müller–Hinton (MH) media (Oxoid, Hampshire, UK) to OD_600_ = 0.1 and then 100×. To each well, 150 µL of the diluted bacterial suspension was pipetted. The inoculated microtitration plates were incubated 24 h at 37 °C. Subsequently, to each well, 10 µL of 10× diluted resazurin indicator Presto Blue (Thermofisher Scientific) was added. Samples were incubated for 0.5 h, abd the absorbance was measured at 570 and 600 nm. The MIC is determined from the difference between the absorbance of the samples at 570 nm and 600 nm.

### 4.11. Viability Testing

Bacterial cultures were incubated in MH (Oxoid, Hampshire, UK) overnight at 37 °C and 150 rpm. The 100 µL of bacterial suspension (~1 × 10^6^ CFU/mL) was placed into 96-well microplate and mixed with EOs-NCaps or EOs in ratio 1:1 (total volume 200 µL). The bacterial growth was detected by Synergy HPX (Biotech). The optical density readouts at 620 nm were monitored at time zero and then at each half-hour for 24 h at 37 °C. The growth rates were calculated according to the following equation:

Growth rate = ((log10 number of cells − log10 number of cells) 2.303)/(time_1_ − time_0_) (6)

The calculated growth rate was related to a positive control (100%).

### 4.12. Haemolytic Assay

The haemolytic assay was done on human erythrocytes. Plasma from the fresh blood sample was collected from laboratory staff. Red blood cells (RBC) were removed by multiple washing steps with 150 mM sodium chloride and centrifugated at 5000 rpm for 5 min. Then, prepared samples were mixed with the RBC and incubated for 1 h at 37 °C. PBS and 0.1% sodium dodecyl suphate (SDS) was used as negative and positive controls, respectively. After completion of the incubation period, the cells were centrifuged and the absorbance of the supernatant containing lysed erythrocytes was measured at 540 nm. The percentage of hemolysis was determined by the following equation:

% Hemolysis = ((A_t_−A_c_)/(A_100%_−A_c_)) × 100 (7)

where A_t_ is the absorbance of the supernatant from samples incubated with the particles, A_c_ is the absorbance of the supernatant from negative control (PBS) and A_100%_ is the absorbance of the positive control supernatant.

### 4.13. Data Treatment and Descriptive Statistics

The experimental work was carried out in the three independent experiments. Obtained data are presented as an average value. Results were analyzed using ANOVA and Scheffe’s Test. A significant result is considered at *p* < 0.05. Data were processed using MICROSOFT EXCEL^®^ (USA).

## 5. Conclusions

The present study was designed to determine the effect of various polysaccharides in C-NCaps on EOs pH-responsive release. The findings of this study suggest that C-G NCaps are suitable for pH-dependent release and show low inhibitory activity on *S. cerevisiae* and antibacterial properties against *S. aureus* and *E. coli*. Other polysaccharide variants showed weaker effects: C-P, C-A, C and C-X, respectively. These results are the first study to compare the properties of different bio nanocapsules. Understanding these mechanisms has the potential to develop NCaps for targeted delivery to the small intestine, where the effects of concentrated antimicrobials are desirable. The synthesis and design of biopolymer carriers have great potential and need to be studied comprehensively. In silico studies would certainly also find application here. Further research should be done to investigate the effect of other antibacterial substances such as metal ions (especially zinc and copper) as well as to clarify the role of other promising polysaccharides for intestinal delivery.

## Abbreviations

A	Alginate
C	Chitosan
C-A NCaps	Chitosan-alginate nanocapsules
C-G NCaps	Chitosan-Guar gum nanocapsules
C-NCaps	Chitosan nanocapsules
C-P NCaps	Chitosan-pectin nanocapsules
C-X NCaps	Chitosan-xanthan nanocapsules
DMSO	Dimethyl sulfoxide
EE	encapsulation efficiency
EOs	Essential oils
FC	Folin-Ciocalteau
G	Guar gum
LC	loading efficiency
MH	Müller–Hinton medium
NCaps	Nanocapsules
P	Pectin
PBS	Phosphate buffer saline
RBC	Red blood cells
R-EOs	*Rosmarinus officinalis* EOs
SDS	Sodium dodecyl sulphate
S-EOs	*Syzygium aromaticum* EOs
T-EOs	*Thymus vulgaris* EOs
TPP	Tripolyphosphate penta basic
X	Xanthan gum
